# In vitro evaluation of the tuberculocidal property of essential oils

**DOI:** 10.1186/1753-6561-5-S6-P42

**Published:** 2011-06-29

**Authors:** S Dharan, G Rajasekaram, P Kaniappan, P Holzner, D Pittet

**Affiliations:** 1 Infection Control Programme, University of Geneva Hospitals, Geneva, Switzerland; 2 Dept of Microbiology, Hospital Sultanah Aminah, Johore Baru, Malaysia; 3 Gold Princess, Geneva, Switzerland

## Introduction / objectives

The re-emergence of tuberculosis on a global scale, together with the emergence and spread of *Mycobacterium tuberculosis* multidrug-resistant strains, is a worldwide public health problem that places a heavy burden on resource-poor countries. We investigated the antibacterial properties of certain essential oils against *M. tuberculosis*.

## Methods

Laboratory tests were carried out in two phases. Phase I was conducted at the Sultanah Aminah Hospital, Malaysia, and phase II at the University of Geneva Hospitals. In phase I, 100 µl of different essential oils were run down the middle of freshly inoculated L-J slants with *M. tuberculosis* to test for growth inhibition by direct contact with the oils. In phase II, we prepared two formulations from the essential oils showing tuberculocidal properties. Actively-growing *M. tuberculosis* cultures were exposed to aerosols with different concentrations of the formulations. After exposure of 5 min daily for 10 days, cultures were incubated for a further 10 days for visual observation of colony growth, followed by subcultures incubated for 6 weeks to evaluate the bactericidal effect.

## Results

Phase I identified 3 oils with a cidal effect on direct contact. In phase II, we identified that a 20% mixture of essential oils in 30% ethanol was tuberculocidal. Subcultures showed no growth up to 3 weeks compared to controls, but showed growth of a few colonies at 6 weeks.

## Conclusions

*In vitro* testing allowed to confirm that certain essential oils have tuberculocidal properties. Inhalation therapy with essential oils could be used as an adjunctive low-cost therapy to directly observed therapy and should be evaluated in controlled clinical trials.

## Disclosure of interest

None declared.

